# Non-parental Childcare During Early Childhood and Problem Behaviour Trajectories from Ages 5 to 14 Years

**DOI:** 10.1007/s10578-024-01703-4

**Published:** 2024-05-14

**Authors:** K. Burdenski, W. Johnson, E. Petherick, S. Costa

**Affiliations:** 1 School of Sport, Exercise and Health Sciences, Loughborough University, Epinal Way, Loughborough, Leicestershire LE11 3TU UK; 2 National Institute for Health Research (NIHR) Leicester Biomedical Research Centre, University Hospitals of Leicester NHS Trust and University of Leicester, Leicester, UK

**Keywords:** Childcare, Early childhood, Mental health, Problem behaviour, Longitudinal

## Abstract

Using data from the nationally representative Millennium Cohort Study, this study examined the association between age of starting and weekly hours in formal childcare between birth and 5 years with internalising and externalising behaviour trajectories from ages 5 to 14 years in England (*N* = 6194 children). Associations were analysed using multilevel general linear regression models, with adjustment for socio-economic position, maternal mental health, demographics, and child temperament. Later entry was associated with more internalising behaviours at age 14 years. Children who spent > 40 h per week in childcare between birth and 3 years displayed more externalising behaviour at 5 years than children who did not attend childcare. Controlling for socio-economic position and parental mental health attenuated findings.

**Supplementary Information:**

The online version contains supplementary material available at 10.1007/s10578-024-01703-4.

## Introduction

Receiving some non-parental childcare has become the norm for children growing up in Western countries [19, 49]. In England, around 90% of children aged 3–4 years and 40% aged 0–2 years attended some formal childcare in 2017 [18]. Children develop through interaction with their environments [9] and early experiences shape long-term mental health. While healthy environments offer opportunities to learn good coping strategies, prosocial skills, and psychological flexibility [5] harmful environments undermine their development and lead to neural changes in stress-related brain areas that have long-term consequences for physical and mental health [46]. Furthermore, childcare exposes children to other adults and children. Children develop through social interaction, and childcare provides them with an opportunity to learn from and form an attachment relationship with another adult [24]. Interactions with other children allow them to develop social skills they could not develop through interactions with adults alone [51]. Given the importance of early childhood experiences and interactions for development, the normalisation of childcare use prompted research and discussions on the association between childcare and psychological development [36]. Studies have reported formal childcare to be related to both better and worse mental health outcomes [8, 16, 51].

Community survey across the world indicate that one third of children will experience a psychological disorder prior to 18 years of age [35] and that 50% of adult psychological disorders have their onset before the age of 14 years [28]. During childhood, psychological problems are traditionally categorised as externalising and internalising [1]. Externalising problems are characterised by aggressiveness, impulsivity and disruptiveness, while internalising problems include anxiety, withdrawal and depression [59]. Both externalising and internalising problems are linked to a reduction in quality of life [44].

Much of the research on the relation between childcare and psychological development has measured internalising and externalising behaviours. The relation between childcare use and internalising and externalising problems appears to be linked to the time spent in childcare, such as the age at which children start attending childcare or the weekly hours they spent in childcare (i.e., intensity). A higher intensity of formal childcare has been associated with higher levels of externalising behaviour [8, 14, 51]. Gupta and Simonsen [25] and Zachrisson et al. [58] reported higher levels of problem behaviour only when formal childcare use exceeded a threshold (30 h and 45 h, respectively), but no difference at lower intensities. Findings for the age of starting childcare are more varied, with both more externalising behaviours [51] and fewer externalising behaviours [16] reported following early commencement of formal childcare.

Favourable outcomes have been reported for internalising behaviour. Higher intensity of formal childcare and an earlier entry have been related to lower levels of internalising and shy behaviour [8, 41]. More time in formal childcare was additionally associated with better social skills [40, 42]. However findings are not universal and many studies also reported non-significant associations of time in formal childcare and internalising and externalising problem behaviours [15, 30].

Studies have analysed the associations of time in early childcare and psychological outcomes in adolescence. Addressing the longevity of associations between childcare and mental health can help understand the potential long-term risks and benefits of attending childcare during a key developmental period in life. Associations reported largely mirror those found at an earlier age, although they appear to become weaker as children age [2, 41]. More time in formal childcare was associated with more externalising behaviour, more impulsivity, and attention deficit hyperactivity disorder symptoms in teenagers (aged 11–15 years) [2, 3, 31, 55]. Pingault et al. [41] reported an association between a higher formal childcare intensity and less shy behaviour at 12 years, while Garon-Carrier and Bégin [20] found no association of any childcare type with depression, anxiety, and disruptive behaviour at 15 years. The association between formal childcare and later problem behaviours may be different depending on the family background [38] and children’s infant emotionality, with infants with a more difficult temperament being more affected by low-quality childcare than children with an easier temperament [4].

Previous studies that explored the association between childcare and adolescent mental health in the UK only measured the type of childcare attended, but not other characteristics of childcare use [31, 38]. Liang et al. [31] explored this in the outer London borough Croydon, while Parkes et al. [38] used a subsample of the nationally representative Millennium Cohort Study (MCS) different to ours. International research findings presented above suggest that the time spent in childcare might also be associated with later mental health. The current study examines the associations between the age of starting and intensity of formal childcare during early childhood and internalising and externalising behaviour trajectories between ages 5 and 14 years in a large and economically diverse sample from England. The study focuses on formal childcare to be comparable to previous research and due to better data quality of formal than informal childcare in the MCS. Using trajectory analyses allows to estimate population-average effects while accounting for the within-subject covariance structure.

## Methods

### Data Set

The MCS is a prospective longitudinal study of 18,819 children born in the UK between September 2000 and January 2002 [13]. A stratified clustered sampling design was used to over-recruit children living in disadvantaged and ethnically diverse neighbourhoods. Interviews with the main caregiver (99.9% the mother in the first sweep) and, where possible, the other parent were carried out by trained interviewers. This study used data from the sweeps 1–6, at ages 9 months, 3, 5, 7, 11, and 14 years [11, 12]. Multicentre Research ethical approval has been received for all sweeps [45]. The present study did not require additional ethics approval as it comprised solely of secondary data analysis of publicly available MCS data. We are grateful to the Centre for Longitudinal Studies, UCL Social Research Institute, for the use of these data and to the UK Data Service for making them available. Neither bear any responsibility for the analysis or interpretation of these data.

### Inclusion and Exclusion Criteria

As all UK countries have slightly different childcare policies, the analysis was limited to children who lived in England during the first three sweeps (8348 children).

Parents needed to have provided information about childcare use in all three sweeps to be eligible for the current study. This could also be information about attending or not attending childcare. Children whose parents provided data that was implausible (e.g., reported an earlier start date than birth date, spent > 70 h per week in childcare) were excluded. Children were excluded when their childcare could not be categorised as formal or informal (the main respondent only reported the use of “other” childcare arrangements) in all sweeps, or when their mothers were childminders looking after their own child.

Children who lived exclusively with their grandparents or foster parents were excluded. In line with previous research [17, 39] and due to different childcare use patterns between singletons and twins [29], twins were excluded from the analysis. Children who were reported to have a disability that limited them in normal activities were also excluded, as children with disabilities have been reported to use childcare differently than children without disabilities [6].

Children needed to have at least one measurement of internalising and externalising behaviour at 5, 7, 11 or 14 years, and have information on all covariates to be included in the analysis.

### Formal Childcare Measures

The childcare variables were derived from data provided at the first three sweeps (9 months, 3 and 5 years). The main respondents were asked what childcare arrangement the child had regularly attended (defined as at least 5 h a week, lasting for at least one month), since when and for how long, at what intensity, and how much it cost. The questions asked about childcare differed at each sweep, with the most comprehensive assessment at the second sweep (including formal and informal childcare, the date of starting and finishing each childcare arrangement, as well as the intensity and cost of those childcare arrangements) (see Jones [27] for a detailed summary of the childcare questionnaires at each sweep). Childminders, nurseries, preschools, and playgroups were classed as formal childcare settings.

#### Age of Starting Childcare

The age children started attending formal childcare was calculated using the reported start date for each childcare arrangement. The earliest reported start date was used, and age of entry was categorised into five groups: younger than 1 year, 1–2 years, 2–3 years, 3–4 years, and 4 years and older.

#### Intensity

Intensity of childcare was defined as the average weekly hours children spent in formal childcare since they first started using it. Two intensity variables were calculated, based on the MCS data structure: one for the time between birth and 3 years, and one for the time between 3 years and school entry, or 3 years and 5 years, whichever came earlier. Two intensity variables were calculated to account for the increased use of childcare for children aged 3 and above compared to younger children in England [26]. Following methods used by Gupta and Simonsen [25], childcare intensity was categorised as: 0 h, 1–10 h, 11–20 h, 21–30 h, 31–40 h, and more than 40 h.

### Outcome Measures

#### Behaviour Problems (Parent-Reported, Sweeps 3–6, Ages 5, 7, 11 and 14 Years)

Externalising and internalising behaviour were assessed with the Strengths and Difficulties Questionnaire (SDQ) [23], a widely used and validated questionnaire of psychopathology symptoms [22]. The main respondent answered 25 items regarding their child’s functioning over the previous 6 months, with 3-point scales ranging from 0 (not true) to 2 (certainly true). The SDQ contains five subscales of five items each, measuring prosocial behaviour, hyperactivity, conduct problems, peer problems and emotional symptoms. In line with best practice for community samples, the hyperactivity and conduct subscales were summed to reflect externalising behaviour and the peer problems and emotional symptoms to reflect internalising behaviour [22]. Scores ranged from 0 to 20, with higher scores indicating more symptoms. The main analysis treated the scores as being on a continuous scale. SDQ scores can be categorised as normal (0–7 for externalising, 0–5 for internalising behaviour), borderline (8–9 for externalising, 6–7 for internalising behaviour), and severe (> 9 for externalising, > 7 for internalising behaviour). These cut-points summed the validated cut-off points for the relevant SDQ components of borderline-abnormal scores [23]. The current research used continuous scores for the SDQ to provide a more nuanced picture of the influence of childcare on problem behaviour than categorical variables would. As this study focused on problem behaviours, the prosocial behaviour subscale was not included. The association between age of starting childcare and prosocial behaviour in the MCS has previously been explored by Peter et al. [40].

### Socio-economic, Child, and Parental Covariates

All covariates were reported at baseline (9 months), so before most children attended any childcare and outcome measures were reported. Thereby, the temporal order of events was respected and the effect of control variables on subsequent change was modelled rather than cross-sectional associations at different time points. Parental caring roles change over time (e.g., through divorce and new partners) so that later SEP variables concerning the partner might be from a different person than at earlier sweeps. Only including control variables at baseline allows to minimise complexities in SEP variables while also keeping the sweep with the least missing data.

Control variables were chosen as we expect them to cause both the exposure and the outcome. Family’s SEP influences their childcare decisions [33], and is also strongly linked with mental health [54]. Maternal mental health and children’s early temperament have also been linked to children’s mental health [32, 50], and might also be linked to the uptake of childcare. The variables were thus all considered potential confounders for the association between early childcare and mental health.

The family’s annual net income was categorised in bands: £0–3100, £3100–£10,400, £10,400–20,800, £20,800–31,200, £31,200–52,000 and £52,000 or more. Mother’s highest qualification according to the English National Qualifications Framework (NQF) was included (GCSE grades D-G, GCSE grade A*–C, A levels, Higher Education Certificate/BTEC (including first degrees and teacher qualifications), and Higher Education Diploma or above (including all postgraduate and higher degrees) [53]. If no information of maternal education was provided, father’s education was used (n = 444). Father’s occupation was classified as unemployed, routine and manual (routine and semi-routine, low supervisory and technical), intermediate (intermediate and small employers and self-employed), and managerial and professional [37]. If no information of father’s occupation was provided, maternal occupation was used (n = 1798). Father’s occupation and maternal education were chosen used so that we have one socio-economic variable for each parent. Given that mothers often take more time off after childbirth, using paternal occupation provided a better proxy for their financial situation, while maternal education is one of the strongest predictors of children’s and adolescents’ mental health [34]. A binary variable indicated whether children lived in a single-parent or dual-parent household. Ethnicity was categorised as White, Mixed, Indian, Pakistani, and Bangladeshi, Black or Black British, and other. A measure of tenure indicated whether families owned the place they lived in (outright or mortgage) or not.

Child gender and parent-rated temperament at 9 months was included. Temperament was assessed with the mood and adaptability to new situations subscales from the Carey Temperament Scale [10]. Parents responded to questions related to their child’s behaviour on a 5-point Likert scale, ranging from almost never (1) to almost always (5). Scores ranged from 5 to 25, with higher values indicating a better mood and more problems adapting to new situations, respectively.

Maternal mental health comprised of two measures: whether they had ever been diagnosed with depression or anxiety, and their overall life satisfaction on a 10-point Likert scale (higher score representing better life satisfaction).

### Statistical Analysis

All statistical analyses were conducted in Stata/MP 17 [47]. Descriptive statistics (frequencies and means and standard deviations) were computed. χ^2^ and t test were performed to compare the sample of this study with the cohort of children excluded from this analysis.

Externalising and internalising behaviour trajectories from 5 to 14 years were modelled in a multilevel general linear regression framework (measurement occasion at level one and individuals at level two) incorporating systematic differences in the sample-average trajectory according to early childcare use and adjustment for covariates. The age scale was centred at 5 years, i.e., the age when children stopped attending childcare. Linear trajectories were produced with a scale of decimal years of age. The constant and slope were allowed to have random effects at level two, with an unstructured variance–covariance matrix. There was a fixed and a random effect for age. The model was used to obtain three key estimates: (1) problem behaviour score at 5 years, (2) annual change in problem behaviour scores between 5 and 14 years (3) problem behaviour scores at 14 years. Age 3 problem behaviour scores were not modelled so that exposure and outcome measures did not overlap in time.

Four analyses were performed for each childcare variable, adjusting for different sets of covariates. Model 1 only included the childcare variable, age, and the age*childcare interaction to obtain the change in problem behaviour associated with childcare attendance. Model 2 adjusted for sex and ethnicity. Model 3 additionally adjusted for the SEP (annual family income, maternal education, paternal occupation, tenure, and number of parents in the household). Model 4 adjusted for the same variables as model 3, while also adjusting for maternal mental health (depression and life satisfaction) and child temperament (mood and adaptability to new situations). All four models were performed on the same sample, i.e. excluding children with missing covariates. For age of starting formal childcare and the intensity of childcare between birth and 3 years, problem behaviour estimates are presented in graphs. Models 1 and 2 and models 3 and 4 were similar, so only the more adjusted of the two was included in the plots. The current analysis did not use a clustered stratified design. The official MCS sample weights adjust for dropouts and ensure a nationally representative analysis, however the current sample only used a subsample of the overall English MCS sample. The weights would therefore not be applicable to the current subsample of the MCS. A variety of socio-economic variables were included to control for possible confounding due to the SEP.

A sensitivity analysis limited to children with externalising and internalising scores at all sweeps was conducted. The sensitivity analysis consisted of a higher SEP subsample. A supplementary analysis of the association between informal childcare use and problem behaviour was conducted, and is presented in Supplementary Material Part 2, Tables SI7 and SI8. Informal childcare was not included in the main analysis as it was not assessed in sweep 3, and we would have only adjusted for a part of informal childcare attendance. Given the heterogeneity of informal childcare, we also had no clear expectation about its influence on the association between formal childcare and problem behaviour.

This study was pre-registered and the code has been published, both can be found under the following URL: https://osf.io/pgs8x/?view_only=cbe349f63512438a87f502ea698d9fb0.

## Results

### Participants

**Fig. 1:**
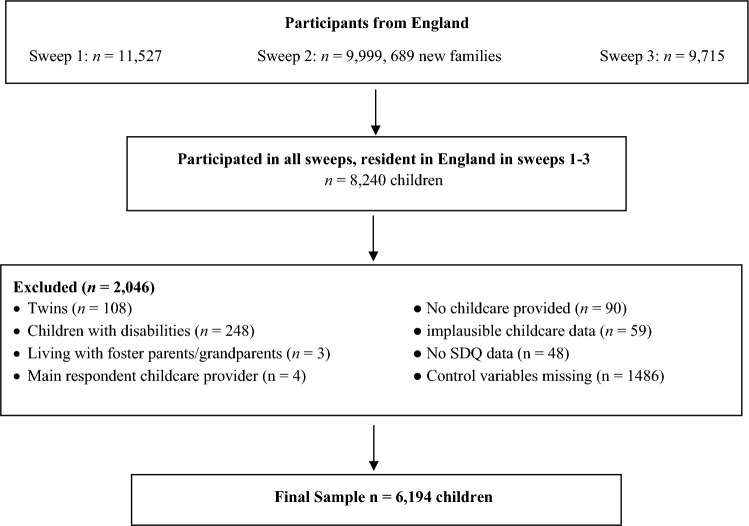
Flow diagram of the sample selection

Six thousand one hundred ninety-four children were included in the analysis, representing 74.2% of MCS children living in England during the first three sweeps. A flow diagram of the sample selection can be found in Fig. 1. The most frequently missing covariates were parental education (*N* = 2330) income (*N* = 1694), maternal life satisfaction (*N* = 1230), and child temperament (*N* = 1184). Descriptive statistics of the sample are presented in Table 1. In addition, Table SI1 in the supplementary material provides the descriptive statistics of the children excluded due to missing covariate data and the differences between the included and excluded sample. Compared to those included in the sample, the excluded children came from lower income families, had fathers with a lower occupational class, and less educated mothers. Excluded children were more likely to be non-White, live in single-parent families, and in rented properties (all *p* < 0.001).

**Table 1:** Description of the participants (*N* = 6194)

Variable	Descriptive statistic
Sex [N (%)]	
Female	3066 (49.5)
Male	3128 (50.5)
Ethnicity [N (%)]	
White	5212 (84.2)
Pakistani and Bangladeshi	315 (5.1)
Indian	183 (3.0)
Black or Black British	180 (2.9)
Mixed	224 (3.6)
Other	80 (1.3)
Annual family income [N (%)]	
£0–3100	75 (1.2)
£3100–10400	1022 (16.5)
£10,400–20800	1987 (32.1)
£20,800–31200	1516 (24.5)
£31,200–52000	1142 (18.4)
£52,000 or more	452 (7.3)
Maternal education [N (%)]	
CGSE grades D–G	639 (10.3)
GCSE grade A*–C	2215 (35.8)
A-level	967 (15.6)
Higher Education Certificate/BTEC	2112 (34.1)
Higher Education Diploma	261 (4.2)
Paternal occupation [N (%)]	
Management and professional	2348 (37.9)
Intermediate	931 (15.0)
Routine and technical	1830 (29.5)
Not working	1085 (17.5)
Parents in household [N (%)]	
Single-parent household	617 (10.0)
Dual-parent household	5577 (90.0)
Tenure [N (%)]	
Own	4197 (67.8)
Do not own	1997 (32.2)
Maternal depression/anxiety [N (%)]	
Yes	1422 (23.0)
No	4772 (77.0)
Maternal life satisfaction [mean (SD]	7.8 (1.7)
Child temperament [mean (SD)]	
Mood	19.2 (3.3)
Adaptability to new situations	9.9 (3.8)
Age of starting formal care [N (%)]	
0–1 year	1148 (31.8)
1–2 years	321 (8.9)
2–3 years	823 (22.8)
3–4 years	1232 (34.1)
4–5 years	85 (2.4)
Intensity of care, birth—3 years [N (%)]	
0 h	3670
1–10 h	766 (37.2)
10–20 h	626 (30.4)
20–30 h	380 (18.5)
30–40 h	197 (9.6)
40+ hours	90 (4.4)
Intensity of care, 3–5 years [N (%)]	
0 h	432
1–10 h	64 (1.2)
10–20 h	3221 (61.9)
20–30 h	960 (18.2)
30–40 h	792 (15.0)
40 + hours	241 (4.6)
Externalising behaviour [mean (SD)]	
5 years	4.5 (3.2)
7 years	4.5 (3.4)
11 years	4.3 (3.4)
14 years	4.1 (3.5)
Internalising behaviour [mean (SD)]	
5 years	2.4 (2.4)
7 years	2.6 (2.7)
11 years	3.1 (3.1)
14 years	3.5 (3.3)

*SD* standard deviation, *GCSE* General Certificate of Secondary Education, *BTEC* Business and Technology Education Council

Entering formal childcare was most common either between 3 and 4 years or in the first year of life. While 35.9% of children attended formal childcare between 0 and 3 years, this increased to 92.4% between 3 and 5 years. Of the children who attended childcare, approximately two thirds did so for less than 20 h and one third for more than 20 h, both before and after 3 years.

On average, externalising behaviour scores slightly decreased with age (4.1 at 14 years and 4.5 at 5 years), while internalising behaviour scores increased (2.4 at 5 years and 3.5 at 14 years). As would be expected for a community sample, the average scores fell into the ‘normal’ range (0–7 for externalising, 0–5 for internalising behaviour). Of all SDQ externalising scores, 83.1% fell into the normal range, 8.2% into the borderline range and 8.7% were classed as severe. Internalising SDQ scores were classed as normal for 84.4% of responses, as borderline for 8.2% and as severe for 7.8% of responses.

### Childcare Use and Externalising and Internalising Behaviour

#### Age of Starting Formal Childcare

**Fig. 2:**
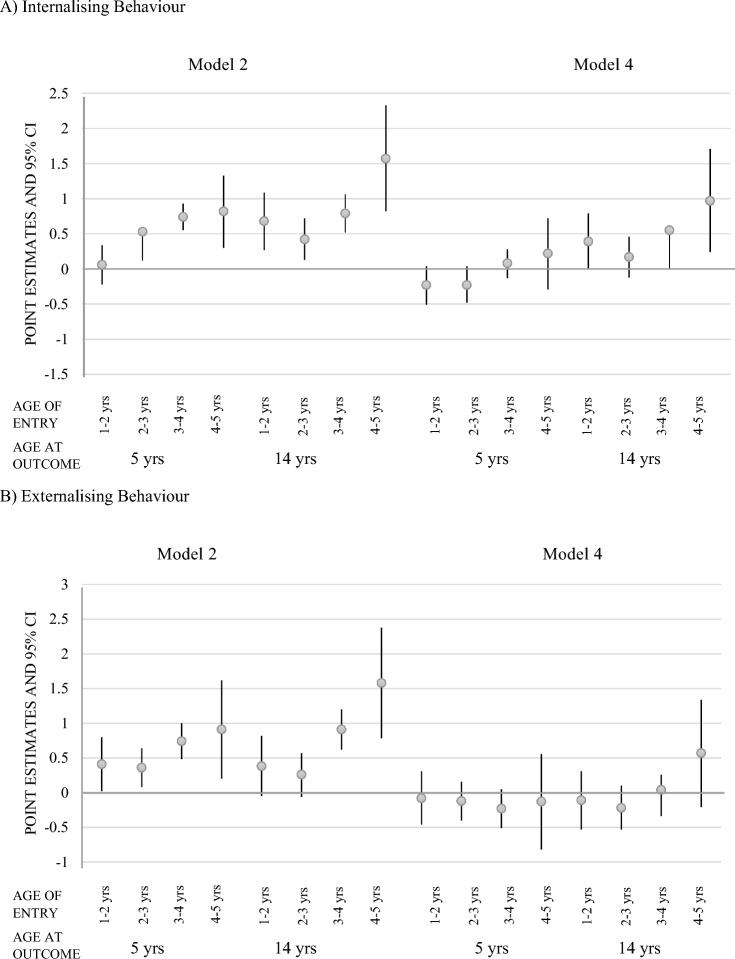
Regression coefficient point estimates and 95% confidence intervals (CI) for the changes in internalising and externalising behaviour scores for children starting childcare at different ages. *Yrs* years

**Fig. 3:**
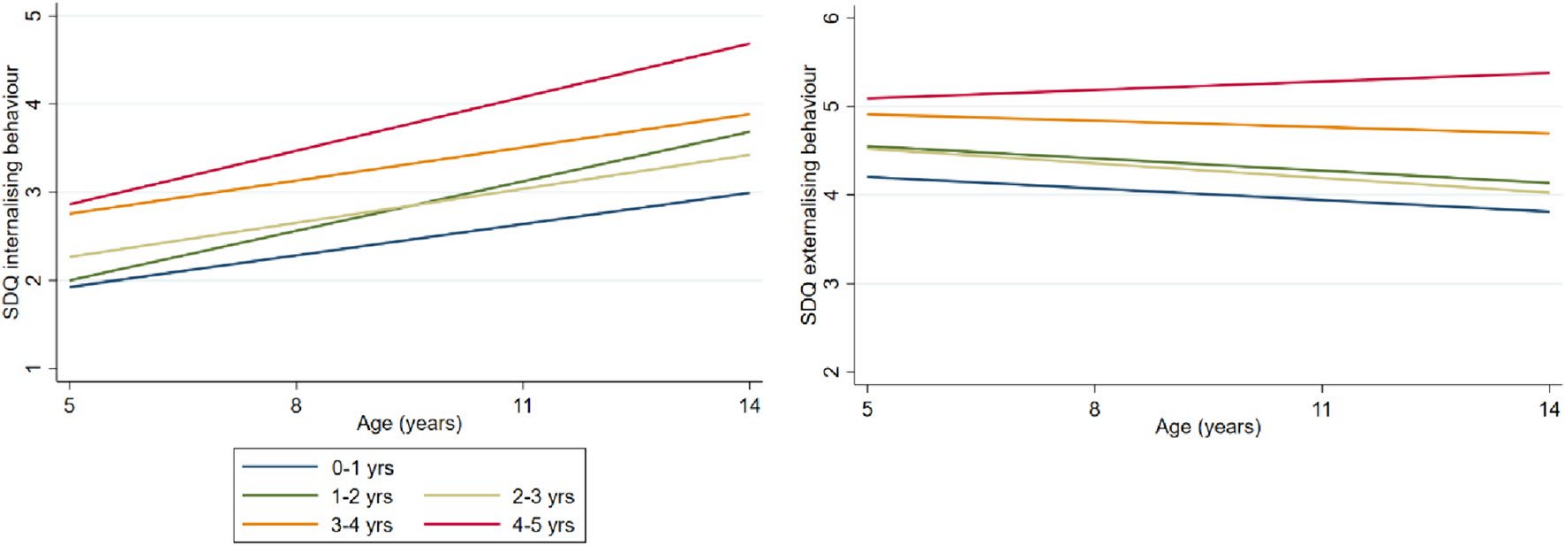
Trajectories for internalising and externalising behaviour scores for children starting childcare at different ages. *Yrs* years

Figure 2 shows the estimated associations of age of starting formal childcare with problem behaviour for two sets of models. Coefficients at ages 5 and 14 are shown as they are the minimum and maximum age for which problem behaviour estimates are presented. Figure 3 shows the SDQ trajectories for children starting childcare at different ages. Parameters for all four models are provided in supplementary information Table SI2. Before adjusting for SEP, starting formal childcare later was associated with more internalising behaviours compared to starting in the first year of life. The associations were stronger at 14 years than at 5 years due to a positive SDQ change coefficient between 5 and 14 years. Adjusting for socio-economic and mental health covariates in models 3 and 4 substantially attenuated estimates. After adjusting for all covariates, starting childcare later compared to in the first year of life was linked to a continuous increase in internalising behaviour SDQ scores at 14 years (*B* = 0.97, *CI* = 0.24–1.71 for children starting childcare between 4 and 5 years).

The pattern was less clear for externalising behaviour. Before controlling for SEP, later entry was associated with more externalising behaviour at 5 years and at 14 years. After adjusting for SEP, the association became negligible. Point estimates were small and mostly negative (ranging from − 0.23 to 0.57), suggesting no association between age of starting childcare and externalising behaviours.

#### Intensity of Formal Childcare

**Fig. 4:**
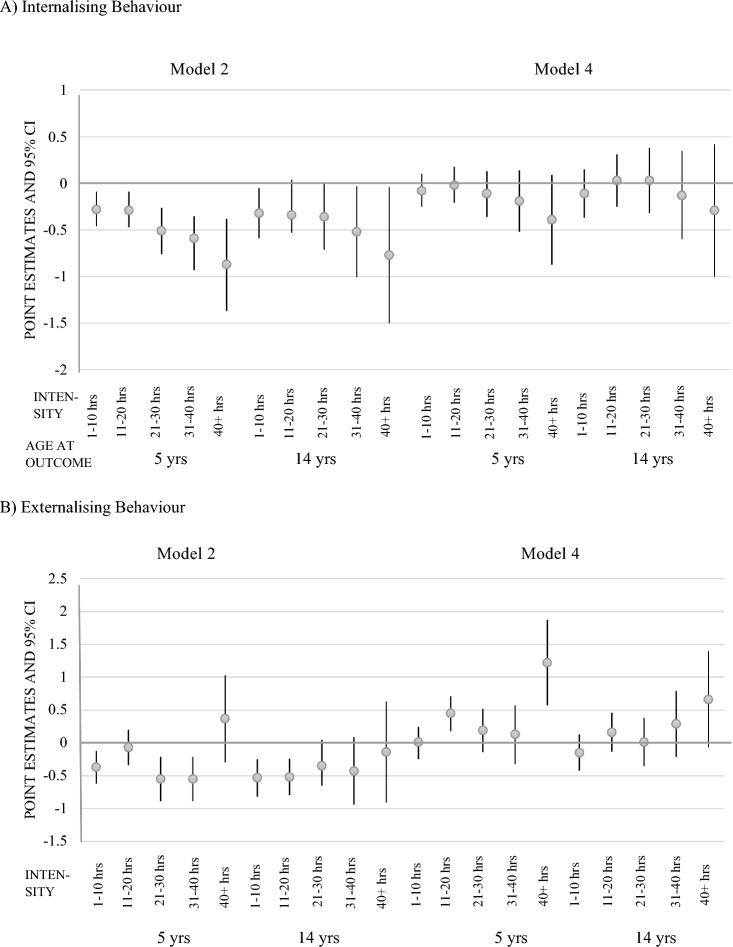
Regression coefficient point estimates and 95% CI for changes in the internalising and externalising behaviour scores for children attending childcare at different intensities between 0 and 3 (referent: no childcare). *Yrs* years, *hrs* hours

**Fig. 5:**
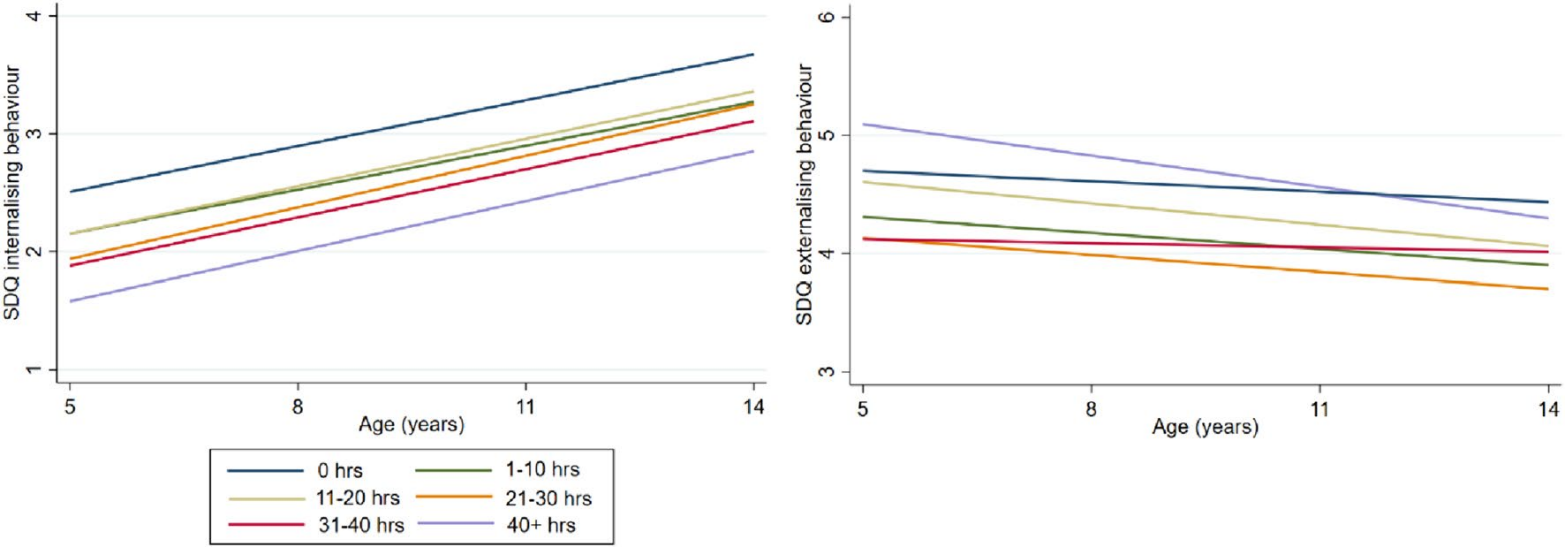
Trajectories for internalising and externalising behaviour scores for children attending childcare at different intensities between 0 and 3 years

Figure 3 shows the estimated association of hours in formal childcare between birth and 3 years and problem behaviour (see Supplementary Information Table SI3 for all parameters) (Fig. 4). The trajectories are shown in Fig. 5. Unadjusted estimates showed that more hours in formal care between 0 and 3 years were related to fewer internalising behaviours at 5 and 14 years. In the fully adjusted model, there was no longer a consistent association between hours in formal care and internalising behaviour.

The associations between externalising behaviour and intensity were less clear. Without adjustment for SEP, most estimates were negative and small, suggesting that more time in childcare was related to less externalising behaviour. After adjusting for all covariates, all childcare intensities were associated with slightly more externalising behaviour at 5 and 14 years. While estimates were negligible for most intensity ranges (i.e., small point estimates with confidence intervals that largely overlap with 0), children who spent 11–20 h displayed slightly more externalising behaviour than children did not attend childcare (*B* = 0.45, *CI* = 0.18–0.71) and children who spent more than 40 h per week in childcare had considerably higher levels of externalising behaviours (*B* = 1.23, *CI* = 0.58–1.87) at 5 years. At 14 years, no meaningful differences in externalising behaviour were measured for the different intensities due to negative SDQ change coefficients.

The intensity of formal childcare between 3 and 5 years was not associated with externalising and internalising behaviour at 5 or 14 years (Supplementary Information Table SI4).

### Sensitivity and Supplementary Analysis

The sensitivity analysis limited to children with externalising and internalising scores at all sweeps (*n* = 4202) yielded comparable associations (Supplementary Information Part 3, Tables SI9–SI1). Informal childcare included care provided by grandparents, relatives (including non-residing parents), friends, neighbourhoods, nannies, or au pairs (see Supplementary Information Part 2, Table SI4 for a description of uptake). Both the age of starting informal childcare and the intensity of informal childcare were not associated with problem behaviours (Supplementary Information Part 2, Tables SI7–SI8).

## Discussion

Using data from a large UK birth cohort study, our results demonstrate a relation between starting formal childcare later and more internalising behaviours at 14 years of age. This was still the case after controlling for SEP, demographics, maternal mental health, and child temperament. Further, there is some evidence suggesting that a higher intensity of formal childcare in the first 3 years of life is associated with more externalising behaviour at 5 years.

An earlier age of starting formal childcare, but not the intensity, was associated with fewer internalising behaviours. The results echo those of Peter et al. [40], who, using MCS data, found that starting childcare before versus after 2.5 years of age was associated with fewer peer problems and better prosocial skills. We extended this research by demonstrating that the association was still visible at 14 years, and by showing that there was a continuous increase in internalising problems with later entry. Importantly, the mental health benefits of starting formal childcare earlier were not apparent right after finishing childcare, suggesting that possible benefits of childcare may become apparent later. A possible contribution to this could be that children acquire better social skills in childcare [40], which may lead to fewer internalising behaviours in later childhood [7]. Children’s internalising scores generally increased between 5 and 14 years, so the increase in estimates may also partly be caused by a greater variability in scores.

Externalising behaviour at 5 years was only related to intensity of formal care for children who spent 11–20 and more than 40 h per week in childcare between 0 and 3 years. With a small point estimate compared to the average externalising score, the clinical significance of the finding for 11–20 h per week in childcare is limited. This initially higher score was followed by a more rapid decrease in externalising behaviour between 5 and 14 years. However, the higher scores for children who spent 40 + hours per week in childcare (1.22 points on the externalising scale, 37.5% of a SD) warrants attention. In line with previous research, this suggests that only children with a very high weekly attendance are at risk for more externalising behaviours [25, 58]. The difference attenuated and was no longer meaningful at 14 years (1.22 vs. 0.66). Furthermore, the results suggest that the timing of exposure matters, as a very high attendance between 3 and 5 was not associated with more externalising behaviours.

All associations became weaker after controlling for SEP, and some flipped directions. The associations initially showed a beneficial relation between more time in childcare and children’s mental health; however, associations became either weaker, non-significant, or negative after controlling for SEP. This suggests that children from a lower SEP who have a higher risk of developing mental health problems [43] attended less childcare. Previous studies suggest that the mental health correlates of childcare differ for children from different SEPs and from adverse home environments [21, 38, 56]. Having fewer children from a lower SEP represented in the higher intensities and earlier start categories might have biased the results to be more applicable to a higher SEP group. When the MCS children were born, all 4-year-old children in England (and some 3-year-old children if born after April 2001 (40.7% of sample)) were entitled to 12.5 h per week of free childcare during 33 weeks annually [57]. All other childcare had to be paid privately. Nowadays, 15 h of free childcare from age 2 years is available for ‘disadvantaged’ children (e.g., families receiving benefits), and families with working parents are entitled to 30 h per week of subsidised childcare from age 3 years [52]. Analysing more recent data with a higher and earlier uptake of low SEP children would allow a more up-to-date understanding of the associations of childcare and mental health.

The pathways through which childcare experiences may affect mental health are poorly understood. The type of childcare has previously been shown to be a determining factor [14, 20, 48]. This was confirmed by our results, showing that informal childcare was not related to children’s problem behaviour (see supplementary material, Sect. 3). The environments provided in formal and informal care are inherently different, most importantly regarding the presence or absence of other children and the qualification of the caregiver. The everyday experience of children looked after by friends and family may be similar to that of being looked after by parents (e.g., fewer other children, more one-to-one interactions), particularly as most informal childcare started in the first year of life. In formal childcare, exposure to other children might promote more acting out (i.e., externalising) behaviour, but also less shy behaviour [51]. As Torres et al. [51] hypothesised, early exposure to peer groups could elicit earlier learning of coercive/dominant strategies for the control of desirable resources. Such behaviours can be perceived as more aggressive, but also characterise normal, friendship-like interactions between children. The results are in line with this explanation. Further investigation with direct observational methods would be required to support such hypotheses.

The implications of childcare attendance for the development of mental illnesses are limited. While, for example, starting childcare between 4 and 5 years may be problematic for the development of internalising behaviours, only 2.4% of children fall in the severe internalising behaviour category. Similarly, only 4.4% of children spent more than 40 h per week in childcare between 0 and 3 years and were at a higher risk for severe externalising behaviours. All other estimates were relatively small and have limited public health significance. Furthermore, many associations explored were non-significant or patterns only emerged for some ages. Moving forward, a more nuanced approach should be taken, combining data on the intensity and age of starting childcare to determine patterns that mirror different user patterns in the ‘real world’. Furthermore, childcare environments vary significantly, and research should explore which childcare characteristics make a setting more advantageous for psychological development (e.g., qualification of staff, activities at childcare). Characteristics that make children susceptible to the childcare experience should also be investigated.

### Strengths and Limitations

This study has expanded previous literature by not only looking at behavioural problems at different ages, but by also analysing trajectories of behavioural problems over time. This allows to better account for within-individual continuity across measurement timepoints. It is the first study to look at the associations of time spent in childcare and internalising behaviour in adolescence, and one of few studies that looked at externalising behaviour measured in adolescence. The wealth of data of the MCS allowed to control for a multitude of socio-economic, child and parental factors that have previously been linked to children’s psychological development and childcare use.

This study has several limitations. Results were not stratified by SEP and socio-economic control variables were only taken from sweep 1 (9 months). A substantial number of cases was missing due to missing covariates, leading to a more socially advantaged sample than the overall MCS sample Future research should specifically focus on the role of SEP in the relationship between childcare and problem behaviour in the UK. We looked at the childcare variables separately and did not consider how informal childcare might have modified the association found for formal childcare. Problem behaviours were only reported by parents and not self-reported or teacher reported, and other outcome measures such as prosocial behaviour and cognitive scores were outside the scope of this research. The analysis adjusted for gender but did not further explore how trends might be different for boys and girls. The sample was limited to England. The estimated associations might be subject to residual confounding. Lastly, quality of childcare was not assessed, and some childcare data could not be included in the parameters as parents provided incomplete data. This likely led to an underestimation of childcare use in the sample.

## Summary

Previous research has shown that more formal childcare in early childhood may be associated with more externalising and less internalising behaviours. There is a lack of research examining this relationship in adolescence in England. This study used data from the nationally representative Millennium Cohort Study to analyse the association between age of starting and weekly hours in formal childcare between birth and 5 years with trajectories of internalising and externalising behaviours between the ages 5–14 years in England (N = 6194 children). Associations were analysed using multilevel general linear regression models, adjusting for demographics, socio-economic position, maternal mental health, and child temperament. The results show an association between later entry into formal childcare and more internalising behaviour at 14 years of age. Very high childcare intensity (> 40 hours per week) between birth and 3 years was associated with more externalising behaviour at 5 years. For all associations, the estimates were small and only few children had clinically high problem behaviour scores, so early formal childcare attendance had limited implications for the development of severe problem behaviours. Controlling for socio-economic position and parental mental health attenuated the results, showing that children from lower socio-economic positions who are at higher risk of developing mental health problems also attended less childcare.

## Conclusion

Later age of starting formal childcare and more hours in childcare were associated with more problem behaviours. Children who started attending childcare later displayed more internalising behaviours at 14 years. Children who attended childcare for more than 40 h per week between birth and 3 years displayed more externalising behaviours. The implications for the development of a severe problem behaviours are limited as estimates are small and only few children fall outside the normal range. Adjusting for SEP significantly attenuated findings or changed the direction of effect, suggesting that children at a higher risk for mental health problem used less childcare.

## Supplementary Information

Below is the link to the electronic supplementary material.

Supplementary file1 (PDF 372 KB)

## Data Availability

The code necessary to reproduce the analyses of this paper is publicly available, and analyses were pre-registered. Both are available at the following URL: https://osf.io/pgs8x/?view_only=cbe349f63512438a87f502ea698d9fb0. The data for this study was downloaded from the UK Data Service and can be found at the following URL: https://beta.ukdataservice.ac.uk/datacatalogue/series/series?id=2000031.
