# Supplementary material for: Non-parental Childcare During Early Childhood and Problem Behaviour Trajectories from Ages 5 to 14 Years
Source: Child Psychiatry Hum Dev. 2024 May 14;57(1):254–66. doi: 10.1007/s10578-024-01703-4 (PMC12971796; doi:10.1007/s10578-024-01703-4)
Supplement: Supplementary file 1 — Supplementary file1 (PDF 372 KB) [file 10578_2024_1703_MOESM1_ESM.pdf]

Supplementary Information

Title: Non-Parental Childcare During Early Childhood and Problem Behaviour Trajectories from 5 to 14 Years

Journal: Child Psychiatry & Human Development

Authors: Burdenski, K., Johnson, W., Petherick, E., & Costa, S.

Table SI1

Comparison and description of the participants included in (*N* = 6,194) and excluded from the sample (*N* = 5,579)

**Variable**	**Descriptive Statistic**		**Difference**
	**Study Sample**	**Excluded participants**	
**Sex (N (%))**			p = .113
Female	3,066 (49.5)	2,343 (48.0)	
Male	3,128 (50.5)	2,540 (52.0)	
**Ethnicity (N (%))**			p < .001
White	5,212 (84.2)	2,918 (60.0)	
Pakistani and Bangladeshi	315 (5.1)	863 (17.8)	
Indian	183 (3.0)	244 (5.0)	
Black or Black British	180 (2.9)	433 (8.9)	
Mixed	224 (3.6)	231 (4.8)	
Other	80 (1.3)	159 (3.3)	
**Annual family income (N (%))**			p < .001
£0-3100	75 (1.2)	112 (2.9)	
£3100-10400	1,022 (16.5)	1,525 (39.3)	
£ 10400-20800	1,987 (32.1)	1,327 (34.2)	
£ 20800-31200	1,516 (24.5)	474 (12.2)	
£31200-52000	1,142 (18.4)	305 (7.9)	
£52000 or more	452 (7.3)	130 (3.4)	
**Maternal Education (N (%))**			p < .001
CGSE grades D- G	639 (10.3)	488 (15.0)	
GCSE grade A*- C	2,215 (35.8)	1,220 (37.6)	
A-level	967 (15.6)	561 (17.3)	
	2,112 (34.1)	853 (26.3)	

Higher Education Certificate/ BTEC Higher Education Diploma	261 (4.2)	127 (3.9)	
**Paternal Occupation (N (%))**			p < .001
Management & professional	2,348 (37.9)	2,106 (43.6)	
Intermediate	931 (15.0)	823 (17.0)	
Routine & technical	1,830 (29.5)	611 (12.7)	
Not working	1,085 (17.5)	1,290 (26.7)	
**Parents in Household (N (%))**			p < .001
Single-parent household	617 (10.0)	1,855 (33.3)	
Dual-parent household	5,577 (90.0)	3,724 (66.8)	
**Tenure (N (%))**			p < .001
Own	4,197 (67.8)	2,033 (42.0)	
Do not own	1,997 (32.2)	2,813 (58.1)	
**Maternal Depression/ Anxiety (N (%))**			p = .003
Yes	1,422 (23.0)	1,001 (20.6)	
No	4,772 (77.0)	3,852 (79.4)	
**Maternal Life Satisfaction (Mean (SD)**			
	7.8 (1.7)	7.6 (2.0)	p < .001
**Child Temperament (Mean (SD)**			
Mood	19.2 (3.3)	18.9 (3.6)	p < .001
Adaptability to new situations	9.9 (3.8)	10.5 (4.2)	p < .001

SD = standard deviation, GCSE = General Certificate of Secondary Education, BTEC = Business and Technology Education Council; * = < .05; ** = <.001

Adjustments in all models

Model 1: not adjusted; Model 2 adjusted for sex and ethnicity; Model 3 adjusted for sex, ethnicity, annual family income, maternal education, paternal occupation, lone parenthood, and tenure. Model 4 adjusted for ethnicity, sex, annual family income, maternal education, paternal occupation, lone parenthood, tenure, maternal depression and life satisfaction and child temperament.

Table SI2

Model parameters for the association between age of entering formal care and problem behaviour.

	Internalising behaviour				Externalising behaviour			
B (95% CI)	Model 1	Model 2	Model 3	Model 4	Model 1	Model 2	Model 3	Model 4
Effect on SDQ at age 5 years								
0-1 years	--	--	--	--	--	--	--	--
1-2 years	0.07 (-0.21-0.36)	0.06 (-0.22-0.34)	-0.20 (-0.48-0.08)	-0.23 (-0.51-0.04)	0.35 (-0.05-0.74)	0.41 (0.02-0.80)	-0.04 (-0.42-0.35)	-0.08 (-0.46-0.31)
2-3 years	0.34 (0.13-0.54)	0.33 (0.12-0.53)	0.06 (-0.15-0.27)	0.08 (-0.13-0.28)	0.32 (0.03-0.61)	0.36 (0.08-0.64)	-0.16 (-0.45-0.12)	-0.12 (-0.40-0.16)
3-4 years	0.83 (0.64-1.01)	0.74 (0.55-0.93)	0.19 (-0.02-0.39)	0.21 (0.01-0.42)	0.71 (0.45-0.97)	0.74 (0.48-1.00)	-0.28 (-0.56--0.00)	-0.23 (-0.51 -0.05)
4-5 years	0.94 (0.42-1.45)	0.82 (0.30-1.33)	0.26 (-0.25-0.77)	0.22 (-0.29-0.72)	0.89 (0.18-1.60)	0.91 (0.20-1.62)	-0.11 (-0.82-0.59)	-0.13 (-0.82-0.56)
Effect on SDQ change per year between 5-14 years								
0-1 years	--	--	--	--	--	--	--	--
1-2 years	0.07 (0.02-0.12)	0.07 (0.02-0.12)	0.07 (0.02-0.12)	0.07 (0.02-0.12)	0.00 (-0.05-0.04)	0.00 (-0.05-0.04)	0.00 (-0.05-0.04)	0.00 (-0.05-0.04)
2-3 years	0.01 (-0.02-0.04)	0.01 (-0.02-0.12)	0.01 (-0.02-0.04)	0.01 (-0.02-0.04)	-0.01 (-0.05-0.02)	-0.01 (-0.05-0.02)	-0.01 (-0.04-0.02)	-0.01 (-0.04-0.02)

3-4 years	0.01 (-0.02-0.04)	0.01 (-0.02-0.04)	0.01 (-0.02-0.04)	0.01 (-0.02-0.04)	0.02 (-0.01-0.05)	0.02 (-0.01-0.05)	0.02 (-0.01-0.05)	0.02 (-0.01-0.05)
4-5 years	0.08 (0.00-0.17)	0.08 (0.00-0.17)	0.08 (0.00-0.17)	0.08 (0.00-0.17)	0.07 (-0.01-0.16)	0.08 (-0.01-0.16)	0.08 (-0.01-0.16)	0.08 (-0.01-0.16)
Effect on SDQ at age 14 years								
0-1 years	--	--	--	--	--	--	--	--
1-2 years	0.69 (0.29-1.10)	0.68 (0.27-1.09)	0.42 (0.02-0.83)	0.39 (-0.01-0.79)	0.32 (-0.11-0.76)	0.38 (-0.05-0.82)	-0.07 (-0.49-0.35)	-0.11 (-0.53-0.31)
2-3 years	0.43 (0.14-0.73)	0.42 (0.13-0.72)	0.16 (-0.15-0.45)	0.17 (-0.12-0.46)	0.21 (-0.10-0.53)	0.26 (-0.06-0.57)	-0.26 (-0.57-0.05)	-0.22 (-0.53-0.10)
3-4 years	0.89 (0.62-1.16)	0.79 (0.52-1.06)	0.25 (-0.04-0.53)	0.28 (0.00-0.55)	0.88 (0.60-1.17)	0.91 (0.62-1.20)	-0.09 (-0.40-0.21)	-0.04 (-0.34-0.26)
4-5 years	1.69 (0.95-2.44)	1.57 (0.82-2.33)	1.02 (0.28-1.77)	0.97 (0.24-1.71)	1.57 (0.76-2.37)	1.58 (0.78-2.38)	0.59 (-0.19-1.37)	0.57 (-0.21-1.34)

Table SI3

Model parameters for the association between the intensity of formal care between 0 and 3 years and problem behaviour.

	Internalising behaviour				Externalising behaviour			
B (95% CI)	Model 1	Model 2	Model 3	Model 4	Model 1	Model 2	Model 3	Model 4
Effect on SDQ at age 5 years								
0	--	--	--	--	--	--	--	--
1-10 hours	-0.36 (-0.54--0.17)	-0.28 (-0.46--0.09)	-0.06 (-0.25-0.12)	-0.08 (-0.25-0.10)	-0.39 (-0.64--0.14)	-0.37 (-0.62--0.12)	0.01 (-0.24-0.25)	0.01 (-0.25-0.24)
11-20 hours	-0.36 (-0.56--0.16)	-0.29 (-0.47--0.09)	0.01 (-0.19-0.21)	-0.02 (-0.21-0.18)	-0.09 (-0.37-0.18)	-0.07 (-0.34-0.20)	0.48 (0.21-0.74)	0.45 (0.18-0.71)
21-30 hours	-0.57 (-0.82--0.32)	-0.51 (-0.76--0.26)	-0.09 (-0.34-0.16)	-0.11 (-0.36-0.13)	-0.57 (-0.91--0.23)	-0.55 (-0.89--0.21)	0.24 (-0.10-0.58)	0.19 (-0.14-0.52)
31-40 hours	-0.63 (-0.97--0.29)	-0.59 (-0.93--0.25)	-0.18 (-0.51-0.16)	-0.19 (-0.52-0.14)	-0.58 (-1.04--0.11)	-0.59 (-1.04--0.13)	0.16 (-0.29-0.61)	0.13 (-0.32-0.57)
40+ hours	-0.93 (-1.42--0.43)	-0.87 (-1.37--0.38)	-0.41 (-0.90-0.08)	-0.39 (-0.87-0.09)	0.40 (-0.28-1.07)	0.37 (-0.30-1.03)	1.23 (0.57-1.89)	1.22 (0.57-1.87)
Effect on SDQ change per year between 5-14 years								
0	--	--	--	--	--	--	--	--
1-10 hours	-0.01 (-0.04-0.03)	0.00 (-0.04-0.03)	0.00 (-0.03-0.03)	0.00 (-0.03-0.03)	-0.02 (-0.05-0.01)	-0.02 (-0.05-0.01)	-0.02 (-0.05-0.01)	-0.02 (-0.05-0.01)
11-20 hours	0.00 (-0.03-0.04)	0.01 (-0.03-0.04)	0.00 (-0.03-0.04)	0.00 (-0.03-0.04)	-0.03 (-0.06-0.00)	-0.03 (-0.06-0.00)	-0.03 (-0.06-0.00)	-0.03 (-0.06-0.00)
21-30 hours	0.02 (-0.02-0.57)	0.02 (-0.02-0.06)	0.02 (-0.02-0.06)	0.02 (-0.02-0.06)	-0.02 (-0.06-0.02)	-0.02 (-0.06-0.02)	-0.02 (-0.06-0.02)	-0.02 (-0.06-0.02)
31-40 hours	0.01 (-0.05-0.06)	0.01 (-0.05-0.06)	0.01 (-0.05-0.06)	0.01 (-0.05-0.06)	-0.02 (-0.04-0.07)	0.02 (-0.04-0.07)	0.02 (-0.04-0.07)	0.02 (-0.04-0.07)

40+ hours	0.01 (-0.07-0.10)	0.01 (-0.07-0.10)	0.01 (-0.07-0.09)	0.01 (-0.07-0.09)	-0.06 (-0.14-0.02)	-0.06 (-0.14-0.02)	-0.06 (-0.14-0.02)	-0.06 (-0.14-0.02)
Effect on SDQ at age 14 years								
0	--	--	--	--	--	--	--	--
1-10 hours	-0.40 (-0.67--0.14)	-0.32 (-0.59--0.05)	-0.10 (-0.37-0.16)	-0.11 (-0.37-0.15)	-0.53 (-0.82--0.25)	-0.52 (-0.80--0.24)	-0.13 (-0.41-0.14)	-0.15 (-0.42-0.13)
11-20 hours	-0.31 (-0.60--0.03)	-0.24 (-0.53-0.04)	0.06 (-0.23-0.34)	0.03 (-0.25-0.31)	-0.37 (-0.68--0.07)	-0.35 (-0.65-0.05)	0.19 (-0.10-0.49)	0.16 (-0.13-0.46)
21-30 hours	-0.42 (-0.78--0.07)	-0.36 (-0.71-0.00)	0.05 (-0.30-0.41)	0.03 (-0.32-0.38)	-0.73 (-1.11--0.36)	-0.72 (-1.09--0.34)	0.06 (-0.31-0.43)	0.01 (-0.35-0.38)
31-40 hours	-0.57 (-1.05--0.08)	-0.52 (-1.01--0.03)	-0.11 (-0.59-0.38)	-0.13 (-0.60-0.35)	-0.42 (-0.94-0.10)	-0.43 (-0.94-0.09)	0.32 (-0.18-0.83)	0.29 (-0.21-0.79)
40+ hours	-0.82 (-1.55--0.09)	-0.77 (-1.50--0.04)	-0.31 (-1.03-0.41)	-0.29 (-1.00-0.42)	-0.14 (-0.91-0.63)	-0.17 (-0.93-0.59)	0.67 (-0.07-1.41)	0.66 (-0.07-1.40)

Table SI4

Model parameters for the association between the intensity of formal care between 3 and 5 years and problem behaviour.

	Internalising behaviour				Externalising behaviour			
B (95% CI)	Model 1	Model 2	Model 3	Model 4	Model 1	Model 2	Model 3	Model 4
Effect on SDQ at age 5 years								
0	--	--	--	--	--	--	--	--
1-10 hours	-0.80 (-1.52- -0.09)	-0.67 (-1.37- 0.04)	-0.35 (-1.05- 0.34)	-0.33 (-1.01- 0.36)	-0.94 (-1.90- 0.02)	-0.90 (-1.85- 0.51)	-0.35 (-1.27- 0.57)	-0.39 (-1.30- 0.53)
10-20 hours	-0.33 (-0.75- 0.09)	-0.21 (-0.63- 0.21)	-0.10 (-0.51- 0.31)	-0.10 (-0.50- 0.31)	-0.55 (-1.12- 0.01)	-0.53 (-1.09- 0.03)	-0.32 (-0.87- 0.22)	-0.33 (-0.87- 0.21)
20-30 hours	-0.48 (-0.92- -0.04)	-0.35 (-0.78- 0.09)	-0.16 (-0.59- 0.27)	-0.17 (0.59- 0.26)	-0.74 (-1.33- -0.15)	-0.72 (-1.31- 0.13)	-0.38 (-0.95- 0.19)	-0.41 (-0.97- 0.15)
30-40 hours	- 0.50 (0.95- -0.06)	-0.39 (-0.83- 0.05)	-0.22 (-0.66- 0.21)	-0.24 (0.67- 0.19)	-0.68 (-1.28- -0.08)	-0.65 (-1.24- -0.06)	-0.35 (-0.93- 0.23)	-0.40 (-0.97- 0.17)
40+ hours	-0.51 (-1.02- 0.02)	-0.37 (-0.88- 0.14)	-0.08 (-0.58- 0.43)	-0.09 (-0.59- 0.40)	-0.47 (-1.16- 0.21)	-0.43 (-1.11- 0.25)	0.09 (-0.57- 0.75)	-0.01 (-0.65- 0.67)
Effect on SDQ change per year between 5-14 years								
0	--	--	--	--	--	--	--	--
1-10 hours	0.09 (-0.03- 0.21)	0.09 (-0.03- 0.21)	0.09 (-0.03- 0.21)	0.09 (-0.03- 0.21)	0.04 (-0.08- 0.15)	0.04 (-0.08- 0.15)	0.03 (-0.08- 0.15)	0.03 (-0.08- 0.15)
10-20 hours	0.04 (-0.03- 0.11)	0.04 (-0.03- 0.11)	0.04 (-0.03- 0.11)	0.04 (-0.03- 0.11)	0.03 (-0.04- 0.10)	0.03 (-0.04- 0.10)	0.03 (-0.04- 0.10)	0.03 (-0.04- 0.10)
20-30 hours	0.03 (-0.04- 0.11)	0.03 (-0.04- 0.11)	0.03 (-0.04- 0.11)	0.03 (-0.04- 0.11)	0.02 (-0.05- 0.10)	0.02 (-0.05- 0.09)	0.02 (-0.05- 0.09)	0.02 (-0.05- 0.09)
30-40 hours	0.06 (-0.01- 0.14)	0.06 (-0.01- 0.14)	0.07 (-0.01- 0.14)	0.07 (-0.01- 0.14)	0.04 (-0.04- 0.11)	0.03 (-0.04- 0.11)	0.04 (-0.04- 0.11)	0.04 (-0.04- 0.11)

40+ hours	0.04 (-0.04-0.12)	0.04 (-0.05-0.12)	0.04 (-0.05-0.12)	0.04 (-0.05-0.13)	0.02 (-0.07-0.10)	0.02 (-0.07-0.10)	0.02 (-0.07-0.10)	0.02 (-0.07-0.10)
Effect on SDQ at age 14 years								
0	--	--	--	--	--	--	--	--
1-10 hours	-0.02 (-1.06-1.01)	0.12 (-0.92-1.16)	0.43 (-0.58-1.45)	0.47 (-0.54-1.47)	-0.59 (-1.69-0.50)	-0.57 (-1.66-0.51)	-0.04 (-1.09-1.01)	-0.08 (-1.12-0.96)
10-20 hours	0.03 (-0.60-0.66)	0.14 (-0.49-0.78)	0.26 (-0.36-0.88)	0.28 (-0.33-0.89)	-0.30 (-0.96-0.37)	-0.29 (-0.95-0.37)	-0.08 (-0.72-0.55)	-0.08 (-0.71-0.55)
20-30 hours	-0.19 (-0.84-0.47)	-0.06 (-0.72-0.60)	0.13 (-0.51-0.78)	0.14 (-0.49-0.78)	-0.54 (-1.23-0.14)	-0.54 (-1.23-0.14)	-0.20 (-0.86-0.46)	-0.22 (-0.88-0.44)
30-40 hours	0.08 (-0.58-0.75)	0.19 (-0.47-0.86)	0.37 (-0.29-1.02)	0.37 (-0.28-1.01)	-0.35 (-1.05-0.35)	-0.34 (-1.03-0.36)	-0.03 (-0.70-0.64)	-0.07 (-0.73-0.60)
40+ hours	-0.17 (-0.92-0.58)	-0.03 (-0.78-0.73)	0.26 (-0.47-1.00)	0.27 (-0.46-1.00)	-0.32 (-1.11-0.47)	-0.29 (-1.07-0.50)	-0.24 (-0.52-1.00)	-0.17 (-0.59-0.93)

Table SI5

Point estimates and confidence intervals of the covariates in the fully adjusted model (Model 4) of the relationship between childcare and problem behaviour.

	Age of Starting Childcare		Intensity 0-3 years		Intensity 3-5 years	
B (95% CI)	Internalising Behaviour	Externalising Behaviour	Internalising Behaviour	Externalising Behaviour	Internalising Behaviour	Externalising Behaviour
Effect on SDQ scores						
**Sex**						
Male	--	--	--	--	--	--
Female	0.02 (-0.11-0.15)	-1.06 (-1.23- -0.88)	-0.01 (-0.12 -0.09)	-1.05 (-1.19- -0.90)	-0.01 (-0.12-0.10)	-1.05 (-1.19- -0.90)
**Ethnicity**						
White	--	--	--	--	--	--
Pakistani/ Bangladeshi	0.54 (-0.19-0.89)	-0.22 (-0.68- 0.24)	0.52 (0.26- 0.78)	-0.14 (-0.49- 0.20)	0.58 (0.32- 0.84)	-0.16 (-0.50- 0.18)
Indian	0.24 (-0.13- 0.60)	0.17 (-0.32- 0.66)	0.26 (-0.07. 0.59)	-0.13 (-0.57-0.30)	0.20 (-0.14- 0.55)	-0.07 (-0.52- 0.38)
Black/ Black British	-0.23 (-0.66-0.20)	-0.78 (-1.35- 0.20)	-0.11 (-0.45- 0.23)	-0.74 (-1.19- -0.29)	-0.06 (-0.43- 0.30)	-0.83 (-1.30- -0.35)
Mixed	-0.02 (-0.38-0.34)	-0.17 (-0.66- 0.32)	-0.07 (-0.37- 0.22)	-0.30 (-0.69- 0.09)	-0.06 (-0.37- 0.25)	-0.22 (-0.62- 0.19)
Other	0.29 (-0.29-0.87)	-0.03 (-0.80- 0.75)	0.09 (-0.39- 0.58)	-0.31 (-0.95 - 0.33)	0.32 (-0.19-0.83)	-0.33 (-1.00 – 0.33)
**Income**						
0-3,100	--	--	--	--	--	--
3,100-10,400	-0.08 (-0.67- 0.52)	0.36 (-0.44- 1.15)	-0.09 (-0.61- 0.43)	0.64 (-0.06- 1.32)	-0.13 (-0.67- 0.42)	0.65 (-0.06-1.36)
10,400-20,800	-0.45 (-1.04- 0.13)	0.42 (-0.36- 1.21)	-0.44 (-0.96- 0.07)	0.32 (-0.36- 1.01)	-0.40 (-0.94- 0.14)	0.40 (-0.31- 1.10)
20,800-31,200	-0.76 (-1.35- -0.17)	0.21 (-0.59-1.01)	-0.73 (-1.25- -0.20)	0.15 (-0.55- 0.84)	-0.70 (-1.25- -0.16)	0.24 (-0.48- 0.95)
31,200-52,000	-0.82 (-1.42- -0.22)	-0.06 (-0.87- 0.75)	-0.74 (-1.27- -0.20)	-0.14 (-0.84- 0.57)	-0.71 (-1.26- -0.16)	-0.06 (-0.78-0.67)
52,000 or more	-1.03 (-1.66- -0.40)	-0.03 (-0.88- 0.82)	-1.00 (-1.56- -0.44)	-0.12 (-0.86-0.62)	-0.96 (-1.54- -0.38)	0.04 (-0.73-0.80)
**Paternal Occupation**						
Not working	--	--	--	--	--	--
Routine/ technical	-0.17 (-0.43- 0.09)	-0.64 (-0.99- -0.29)	-0.19 (-0.40- 0.01)	-0.44 (-0.71- -0.17)	-0.19 (-0.40- 0.03)	-0.43 (-0.71- -0.15)
Intermediate	0.03 (-0.25- 0.31)	-0.45 (-0.82-0.07)	-0.13 (-0.35- 0.08)	-0.25 (-0.54- 0.04)	-0.14 (-0.36- 0.09)	-0.22 (-0.51- 0.08)
Management/ professional	0.19 (-0.06- 0.43)	0.03 (-0.29-0.36)	0.14 (-0.06- 0.33)	0.18 (-0.07- 0.43)	0.11 (-0.09- 0.31)	0.14 (-0.12- 0.39)
**Maternal Education**						
GCSE grades D-G	--	--	--	--	--	--
GCSE grade A*-C	-0.19 (-0.44- 0.06)	-0.62 (-0.95- -0.28)	-0.14 (-0.34- 0.05)	-0.34 (-0.60- -0.09)	-0.11 (-0.31- 0.09)	-0.26 (-0.52- 0.00)
A-level	-0.42 (-0.70- 0.13)	-0.90 (-1.28- -0.52)	-0.20 (-0.42-0.02)	-0.59 (-0.88- -0.29)	-0.18 (-0.41- 0.04)	-0.50 (-0.80- -0.21)
Higher Education Certificate/BTEC	-0.29 (-0.55- 0.02)	-1.09 (-1.45- -0.73)	-0.23 (-0.44- -0.02)	-0.86 (-1.14- -0.59)	-0.22 (-0.43- 0.00)	-0.80 (-1.08- -0.53)
Higher Education Diploma	-0.10 (-0.49- 0.29)	-1.25 (-1.77- -0.73)	-0.07 (-0.41- 0.27)	-0.96 (-1.41- -0.51)	-0.09 (-0.44- 0.26)	-0.78 (-1.24- -0.32)

**Parents in household**						
Single-parent household	--	--	--	--	--	--
Dual-parent household	0.00 (-0.29-0.28)	0.31 (-0.07 – 0.69)	-0.02 (-0.25- 0.21)	0.03 (-0.27- 0.34)	0.01 (-0.23- 0.25)	0.07 (-0.24-0.39)
**Tenure**						
Own	--	--	--	--	--	--
Do not own	-0.12 (-0.30-0.07)	-0.57 (-0.81- -0.32)	-0.24 (-0.38- -0.09)	-0.61 (-0.81- -0.42)	-0.26 (-0.41- -0.11)	-0.60 (-0.79- .0.40)
**Maternal Depression/ Anxiety**						
No	--	--	--	--	--	--
Yes	0.35 (0.19- 0.52)	0.34 (0.12-0.56)	0.46 (0.33- 0.59)	0.41 (0.23- 0.58)	0.49 (0.35- 0.62)	0.47 (0.29- 0.65)
**Maternal Life Satisfaction**	-0.17 (-0.22- 0.13)	-0.17 (-0.23- -0.11)	-0.17 (-0.20- -0.13)	-0.18 (-0.23- 0.14)	-0.17 (-0.20- -0.13)	-0.19 (-0.24- -0.15)
**Child temperament: bad mood**	-0.03 (-0.05- -0.01)	-0.06 (-0.09- -0.04)	-0.03 (-0.04- -0.01)	-0.06 (-0.08- -0.04)	-0.02 (-0.04- 0.00)	-0.06 (-0.08- -0.04)
**Child temperament: adaptability**	0.04 (0.03- 0.06)	0.00 (-0.02-0.03)	0.05 (0.03- 0.06)	0.01 (-0.01- 0.03)	0.05 (0.04-0.07)	0.01 (-0.01-0.03)

Part 2. Supplementary Analysis of Informal Childcare Use

Measures of Informal Care

Supplementary analyses explored associations between informal childcare use and children's problem behaviours. Care provided by grandparents, relatives (including non-residing parents), friends, neighbourhoods, nannies, or au pairs was classed as informal. Data on informal childcare was only available at the first two sweeps so that the intensity of informal care was only calculated for ages 0 to 3 years. Age of starting informal childcare entry was categorised as younger than 1 year, 1 to 2 years, and 2 years and older.

Statistical Analysis

Frequencies of childcare use were calculated. The same multilevel general linear regression models were used for informal care as for formal care. For each childcare exposure and outcome variable, four models were calculated with different sets of covariates (model 1: no covariate; model 2: sex and ethnicity; model 3: sex, ethnicity, and SEP (annual family income, maternal education, paternal occupation, tenure and number of parents in the household); model 4: sex, ethnicity, SEP, maternal mental health (depression and life satisfaction) and child temperament). See the main paper's method section for a more detailed description of the analysis.

Results

Table SI4 summarises the use of informal childcare in the sample. Most children started attending informal childcare in the first year of life. Most children were cared for by an informal caregiver for 10-20 hours or 1-10 hours.

Table SI6

Description of Informal Childcare Use

	N	%
**Age of Starting Care**		
0-1 year	1601	82.5
1-2 years	177	9.1
2 years +	162	8.4
**Intensity of Care, birth- 3 years**		
0 hours	4204	-
1-10 hours	452	28.7
10-20 hours	568	36.0
20-30 hours	306	19.4
30-40 hours	177	11.2
40 hours +	74	4.7

Tables SI5 and SI6 provide the model parameters for the association between childcare use and internalising and externalising behaviour scores in the sample. After controlling for SEP and mental health, both the age of starting informal childcare and the intensity of informal childcare were not associated with problem behaviours.

Table SI7

Model parameters for the association between age of entering informal care and problem behaviour.

	Internalising behaviour				Externalising behaviour			
B (95% CI)	Model 1	Model 2	Model 3	Model 4	Model 1	Model 2	Model 3	Model 4
Effect on SDQ at age 5 years								
0-1 years	--	--	--	--	--	--	--	--
1-2 years	-0.08 (-0.43-0.27)	-0.07 (-0.42-0.28)	-0.23 (-0.57-0.11)	-0.29 (-0.63-0.05)	-0.16 (-0.65-0.34)	-0.12 (-0.61-0.38)	-0.35 (-0.83-0.13)	-0.38 (-0.86-0.09)
2-4 years	0.16 (-0.21-0.52)	0.18 (-0.18-0.54)	0.00 (-0.35-0.36)	0.01 (-0.34-0.36)	0.06 (-0.45-0.58)	0.10 (-0.41-0.61)	-0.19 (-0.69-0.31)	-0.17 (-0.66-0.33)
Effect on SDQ change per year between 5-14 years								
0-1 years	--	--	--	--	--	--	--	--
1-2 years	0.06 (0.00-0.11)	0.06 (0.00-0.11)	0.05 (0.00-0.11)	0.05 (0.00-0.11)	0.01 (-0.05-0.07)	0.01 (-0.05-0.07)	0.01 (-0.05-0.07)	0.01 (-0.05-0.07)
2-4 years	0.06 (-0.01-0.12)	0.06 (-0.01-0.12)	0.06 (-0.01-0.12)	0.06 (-0.01-0.12)	0.05 (-0.02-0.11)	0.05 (-0.01-0.11)	0.05 (-0.01-0.12)	0.05 (-0.01-0.12)
Effect on SDQ at age 14 years								
0-1 years	--	--	--	--	--	--	--	--
1-2 years	0.42 (-0.09-0.93)	0.43 (-0.08-0.95)	0.27 (-0.24-0.77)	0.19 (-0.31-0.69)	-0.05 (-0.62-0.52)	-0.02 (-0.58-0.55)	-0.26 (-0.81-0.28)	-0.30 (-0.85-0.24)
2-4 years	0.66 (0.11-1.21)	0.68 (0.13-1.23)	0.51 (-0.03-1.06)	0.52 (-0.01-1.06)	0.50 (-0.11-1.11)	0.54 (-0.06-1.15)	0.28 (-0.30-0.86)	0.31 (-0.27-0.89)

Table SI8

Model parameters for the association between the intensity of informal care between 0 and 3 years and problem behaviour.

	Internalising behaviour				Externalising behaviour			
B (95% CI)	Model 1	Model 2	Model 3	Model 4	Model 1	Model 2	Model 3	Model 4
Effect on SDQ at age 5 years								
0	--	--	--	--	--	--	--	--
1-10 hours	0.06 (-0.17-0.29)	0.09 (-0.14-0.31)	0.06 (-0.16-0.29)	0.12 (-0.10-0.35)	0.10 (-0.21-0.41)	0.07 (-0.24-0.38)	0.03 (-0.27-0.33)	0.09 (-0.20-0.39)
11-20 hours	-0.30 (-0.51--0.09)	-0.26 (-0.46-0.05)	-0.18 (-0.38-0.03)	-0.14 (-0.34-0.06)	-0.14 (-0.42-0.14)	-0.12 (-0.39-0.16)	0.01 (-0.26-0.28)	0.05 (-0.22-0.32)
21-30 hours	-0.25 (-0.52-0.03)	-0.22 (-0.49-0.05)	-0.15 (-0.41-0.12)	-0.06 (-0.32-0.21)	-0.12 (-0.49-0.25)	0.11 (-0.48-0.26)	-0.05 (-0.41-0.31)	0.04 (-0.32-0.39)
31-40 hours	-0.06 (-0.41-0.30)	-0.04 (-0.40-0.31)	0.08 (-0.27-0.43)	0.12 (-0.22-0.47)	0.30 (-0.18-0.79)	0.30 (-0.18-0.78)	0.46 (0.01-0.93)	0.50 (0.04-0.97)
40+ hours	-0.34 (-0.88-0.20)	-0.32 (-0.86-0.21)	-0.17 (-0.70-0.35)	-0.13 (-0.65-0.39)	0.14 (-0.60-0.88)	0.12 (-0.62-0.85)	0.28 (-0.43-0.99)	0.32 (-0.38-1.03)
Effect on SDQ change per year between 5-14 years								
0	--	--	--	--	--	--	--	--
1-10 hours	0.02 (-0.02-0.06)	0.02 (-0.02-0.06)	0.02 (-0.02-0.06)	0.02 (-0.02-0.06)	0.00 (-0.04-0.03)	0.00 (-0.04-0.03)	0.00 (-0.04-0.03)	0.00 (-0.04-0.03)
11-20 hours	0.00 (-0.03-0.03)	0.00 (-0.03-0.03)	0.00 (-0.03-0.04)	0.00 (-0.03-0.03)	-0.01 (-0.05-0.02)	-0.01 (-0.05-0.02)	-0.01 (-0.05-0.02)	-0.01 (-0.05-0.02)
21-30 hours	0.00 (-0.04-0.05)	0.00 (-0.04-0.05)	0.00 (-0.04-0.05)	0.00 (-0.04-0.05)	-0.02 (-0.07-0.02)	-0.02 (-0.07-0.02)	-0.02 (-0.07-0.02)	-0.02 (-0.07-0.02)
31-40 hours	0.00 (-0.06-0.06)	0.00 (-0.06-0.06)	0.00 (-0.06-0.06)	0.00 (-0.06-0.06)	-0.01 (-0.07-0.05)	-0.01 (-0.07-0.05)	-0.01 (-0.07-0.05)	-0.01 (-0.07-0.05)

40+ hours	0.08 (-0.01-0.17)	0.08 (-0.01-0.17)	0.08 (-0.01-0.18)	0.08 (-0.01-0.18)	0.02 (-0.07-0.11)	0.02 (-0.07-0.10)	0.02 (-0.07-0.11)	0.02 (-0.07-0.11)
Effect on SDQ at age 14 years								
0	--	--	--	--	--	--	--	--
1-10 hours	0.24 (-0.09-0.57)	0.27 (-0.07-0.60)	0.24 (-0.08-0.57)	0.30 (-0.02-0.63)	0.07 (-0.28-0.42)	0.04 (-0.31-0.39)	0.00 (-0.33-0.34)	0.07 (-0.27-0.40)
11-20 hours	-0.30 (-0.60-0.00)	-0.26 (-0.56-0.05)	-0.17 (-0.47-0.13)	-0.14 (-0.43-0.16)	-0.27 (-0.59-0.04)	-0.25 (-0.56-0.07)	-0.11 (-0.42-0.19)	-0.08 (-0.38-0.22)
21-30 hours	-0.23 (-0.63-0.17)	-0.20 (-0.60-0.20)	-0.13 (-0.52-0.26)	-0.04 (-0.43-0.35)	-0.33 (-0.75-0.09)	-0.32 (-0.74-0.09)	-0.27 (-0.67-0.14)	-0.18 (-0.58-0.22)
31-40 hours	0.06 (-0.57-0.46)	-0.04 (-0.56-0.48)	0.08 (-0.42-0.59)	0.12 (-0.38-0.63)	0.24 (-0.31-0.78)	0.23 (-0.31-0.77)	0.40 (-0.13-0.92)	0.44 (-0.08-0.96)
40+ hours	0.40 (-0.39-1.19)	0.42 (-0.37-1.22)	0.59 (-0.19-1.37)	0.63 (-0.14-1.40)	0.28 (-0.55-1.12)	0.25 (-0.58-1.08)	0.45 (-0.35-1.25)	0.48 (-0.31-1.28)

Part 3. Sensitivity analysis with children who have SDQ data at all sweeps (*n* = 4,202)

Table SI9

Model parameters for the association between age of entering formal care and problem behaviour.

	Internalising behaviour				Externalising behaviour			
B (95% CI)	Model 1	Model 2	Model 3	Model 4	Model 1	Model 2	Model 3	Model 4
Effect on SDQ at age 5 years								
0-1 years	--	--	--	--	--	--	--	--
1-2 years	0.04 (-0.28-0.35)	0.03 (-0.28-0.35)	-0.25 (-0.56-0.07)	-0.27 (-0.58-0.04)	0.21 (-0.24-0.66)	0.26 (-0.19-0.70)	-0.19 (-0.63-0.25)	-0.21 (-0.65-0.22)
2-3 years	0.29 (0.06-0.53)	0.30 (0.07-0.53)	0.04 (-0.20-0.27)	0.06 (-0.17-0.29)	0.38 (0.05-0.71)	0.43 (0.11-0.76)	-0.02 (-0.35-0.31)	0.03 (-0.29-0.35)
3-4 years	0.71 (0.50-0.93)	0.65 (0.43-0.87)	0.11 (-0.13-0.35)	0.14 (-0.09-0.38)	0.66 (0.36-0.97)	0.63 (0.33-0.94)	-0.27 (0.59-0.06)	-0.20 (-0.53-0.12)
4-5 years	0.46 (-0.16-1.08)	0.35 (-0.27-0.98)	-0.18 (-0.81-0.44)	-0.16 (-0.78-0.46)	0.99 (0.10-1.87)	0.94 (0.06-1.81)	0.02 (-0.85-0.88)	0.03 (-0.82-0.89)
Effect on SDQ change per year between 5-14 years								
0-1 years	--	--	--	--	--	--	--	--
1-2 years	0.07 (0.02-0.11)	0.07 (0.02-0.11)	0.06 (0.02-0.11)	0.06 (0.02-0.11)	0.00 (-0.05-0.05)	0.00 (-0.05-0.05)	0.00 (-0.05-0.05)	0.00 (-0.05-0.05)
2-3 years	0.02 (-0.02-0.05)	0.02 (-0.02-0.05)	0.02 (-0.02-0.05)	0.02 (-0.02-0.05)	-0.02 (-0.05-0.02)	-0.01 (-0.05-0.02)	-0.01 (-0.05-0.02)	-0.01 (-0.05-0.02)
3-4 years	0.03 (-0.01-0.06)	0.03 (-0.01-0.06)	0.03 (-0.01-0.06)	0.03 (-0.01-0.06)	0.03 (0.00-0.07)	0.03 (0.00-0.07)	0.03 (0.00-0.07)	0.03 (0.00-0.07)
4-5 years	0.12 (0.03-0.22)	0.12 (0.03-0.22)	0.12 (0.03-0.22)	0.12 (0.03-0.22)	0.07 (-0.02-0.17)	0.07 (-0.02-0.17)	0.07 (-0.02-0.17)	0.07 (-0.02-0.17)

Effect on SDQ at age 14 years								
0-1 years	--	--	--	--	--	--	--	--
1-2 years	0.62 (0.19-1.06)	0.62 (0.18-1.06)	0.34 (-0.10-0.77)	0.32 (-0.11-0.74)	0.24 (-0.24-0.71)	0.29 (-0.18-0.76)	-0.16 (-0.61-0.30)	-0.18 (-0.64-0.27)
2-3 years	0.44 (0.12-0.76)	0.44 (0.12-0.77)	0.19 (-0.13-0.51)	0.21 (-0.11-0.53)	0.26 (-0.09-0.60)	0.31 (-0.03-0.66)	-0.14 (-0.48-0.20)	-0.09 (-0.43-0.25)
3-4 years	0.96 (0.66-1.26)	0.90 (0.60-1.20)	0.36 (0.05-0.67)	0.39 (0.09-0.70)	0.97 (0.65-1.30)	0.94 (0.62-1.27)	0.04 (-0.29-0.38)	0.11 (-0.23-0.45)
4-5 years	1.55 (0.70-2.40)	1.45 (0.59-2.30)	0.91 (0.07-1.75)	0.93 (0.10-1.77)	1.64 (0.71-2.56)	1.59 (0.67-2.51)	0.66 (-0.23-1.56)	0.68 (-0.21-1.57)

Table SI10

Model parameters for the association between the intensity of formal care between 0 and 3 years and problem behaviour.

	Internalising behaviour				Externalising behaviour			
B (95% CI)	Model 1	Model 2	Model 3	Model 4	Model 1	Model 2	Model 3	Model 4
Effect on SDQ at age 5 years								
0	--	--	--	--	--	--	--	--
1-10 hours	-0.29 (-0.49- -0.08)	-0.22 (-0.43- -0.02)	-0.02 (-0.23- 0.19)	-0.04 (-0.24- 0.17)	-0.39 (-0.68- -0.10)	-0.35 (-0.63 - 0.06)	-0.02 (-0.30- 0.27)	-0.04 (-0.32- 0.24)
11-20 hours	-0.34 (-0.56- -0.12)	-0.29 (-0.51- -0.08)	-0.01 (-0.23- 0.21)	-0.03 (-0.25- 0.18)	-0.13 (-0.44- 0.17)	-0.10 (-0.41- 0.20)	0.35 (0.05- 0.65)	0.33 (0.03- 0.63)
21-30 hours	-0.48 (-0.75- -0.21)	-0.43 (-0.71- -0.16)	-0.02 (-0.29- 0.26)	-0.05 (-0.33- 0.22)	-0.53 (-0.91- -0.15)	-0.50 (-0.88- -0.13)	0.23 (-0.15- 0.61)	0.18 (-0.20- 0.55)
31-40 hours	-0.52 (-0.90- -0.14)	-0.50 (-0.88- -0.12)	-0.09 (-0.47- 0.29)	-0.13 (-0.50- 0.25)	-0.47 (-1.00- 0.06)	-0.47 (-0.99- 0.06)	0.21 (-0.31- 0.73)	0.17 (-0.35- 0.68)
40+ hours	-0.85 (-1.43- -0.27)	-0.83 (-1.40- -0.25)	-0.40 (-0.98- 0.17)	-0.38 (0.94- 0.18)	0.25 (-0.55- 1.06)	0.22 (-0.58- 1.01)	0.90 (0.12- 1.68)	0.90 (0.12- 1.67)
Effect on SDQ change per year between 5-14 years								
0	--	--	--	--	--	--	--	--
1-10 hours	0.00 (-0.04- 0.03)	0.00 (-0.04- 0.03)	0.00 (-0.04- 0.03)	0.00 (-0.04- 0.03)	-0.02 (-0.06- 0.01)	-0.02 (-0.05- 0.01)	-0.02 (-0.05- 0.01)	-0.02 (-0.05- 0.01)
11-20 hours	0.00 (-0.03- 0.04)	0.00 (-0.03- 0.04)	0.00 (-0.03- 0.04)	0.00 (-0.03- 0.04)	-0.03 (-0.06- 0.00)	-0.03 (-0.06- 0.00)	-0.03 (-0.06- 0.00)	-0.03 (-0.06- 0.00)
21-30 hours	0.00 (-0.05- 0.04)	0.00 (-0.05- 0.04)	0.00 (-0.05- 0.04)	0.00 (-0.05- 0.04)	-0.04 (-0.08- 0.00)	-0.04 (-0.08- 0.00)	-0.04 (-0.08- 0.00)	-0.04 (-0.08- 0.00)
31-40 hours	0.01 (-0.05- 0.07)	0.01 (-0.05- 0.07)	0.01 (-0.05- 0.07)	0.01 (-0.05- 0.07)	0.01 (-0.05- 0.07)	0.01 (-0.05- 0.07)	0.01 (-0.05- 0.07)	0.01 (-0.05- 0.07)

40+ hours	-0.02 (-0.11-0.07)	-0.02 (-0.11-0.07)	-0.02 (-0.11-0.07)	-0.02 (-0.11-0.07)	-0.04 (-0.13-0.05)	-0.04 (-0.13-0.05)	-0.04 (-0.13-0.05)	-0.04 (-0.13-0.05)
Effect on SDQ at age 14 years								
0	--	--	--	--	--	--	--	--
1-10 hours	-0.32 (-0.61--0.03)	-0.26 (-0.54-0.03)	-0.06 (-0.34-0.23)	-0.07 (-0.35-0.21)	-0.57 (-0.88-0.27)	-0.53 (-0.84--0.23)	-0.21 (-0.50-0.09)	-0.23 (-0.52-0.07)
11-20 hours	-0.32 (-0.63--0.02)	-0.28 (-0.58-0.03)	0.01 (-0.30-0.31)	-0.01 (-0.31-0.29)	-0.41 (-0.73 -0.08)	-0.38 (-0.70--0.06)	0.08 (-0.24-0.39)	0.05 (-0.26-0.37)
21-30 hours	-0.51 (-0.89--0.13)	-0.46 (-0.84--0.08)	-0.05 (-0.42-0.33)	-0.08 (-0.46-0.29)	-0.86 (-1.27--0.46)	-0.84 (-1.24--0.44)	-0.10 (-0.50-0.30)	-0.16 (-0.55-0.24)
31-40 hours	-0.44 (-0.96-0.09)	-0.41 (-0.94-0.12)	-0.01 (-0.53-0.51)	-0.04 (0.56-0.48)	-0.39 (-0.95-0.17)	-0.39 (-0.94-0.17)	-0.29 (-0.25-0.84)	0.25 (-0.30-0.79)
40+ hours	-1.01 (-1.81--0.21)	-0.98 (-1.79--0.18)	-0.56 (-1.36-0.23)	-0.54 (-1.32-0.24)	-0.09 (-0.95-0.76)	-0.13 (-0.98-0.72)	0.56 (-0.27-1.38)	0.55 (-0.27-1.37)

Table SI11

Model parameters for the association between the intensity of formal care between 3 and 5 years and problem behaviour.

	Internalising behaviour				Externalising behaviour			
B (95% CI)	Model 1	Model 2	Model 3	Model 4	Model 1	Model 2	Model 3	Model 4
Effect on SDQ at age 5 years								
0	--	--	--	--	--	--	--	--
1-10 hours	-0.88 (-1.71- -0.06)	-0.73 (-1.55- 0.09)	-0.48 (-1.28- 0.32)	-0.47 (-1.26- 0.33)	-0.73 (-1.85- 0.39)	-0.72 (-1.83- 0.38)	-0.35 (-1.43- 0.73)	-0.43 (-1.50- 0.65)
10-20 hours	-0.43 (-0.97- 0.10)	-0.32 (-0.86- 0.22)	-0.23 (-0.76- 0.29)	-0.20 (-0.72- 0.32)	0.55 (-1.28- 0.18)	-0.57 (-1.29- 0.16)	-0.41 (-1.12- 0.29)	-0.40 (-1.10- 0.30)
20-30 hours	-0.52 (-1.07- 0.04)	-0.39 (-0.95- 0.16)	-0.22 (-0.77- 0.32)	-0.21 (-0.75- 0.33)	-0.78 (-1.54- -0.02)	-0.80 (-1.55- -0.05)	-0.52 (-1.25- 0.21)	-0.54 (-1.27- 0.19)
30-40 hours	-0.62 (-1.19- -0.06)	-0.52 (-1.08- 0.05)	(-0.35 (-0.90- 0.20)	-0.33 (-0.88- 0.22)	-0.71 (-1.48- 0.06)	-0.74 (-1.50- 0.02)	-0.46 (-1.20- 0.28)	-0.49 (-1.23- 0.25)
40+ hours	-0.42 (-1.05- 0.22)	-0.29 (-0.92- 0.34)	-0.01 (-0.63- 0.61)	0.02 (-0.59- 0.64)	-0.45 (-1.31- 0.41)	-0.44 (-1.29- 0.42)	0.06 (-0.77- 0.89)	0.00 (-0.82- 0.83)
Effect on SDQ change per year between 5-14 years								
0	--	--	--	--	--	--	--	--
1-10 hours	0.13 (0.01- 0.26)	0.13 (0.01- 0.26)	0.13 (0.01- 0.26)	0.13 (0.01- 0.26)	0.03 (-0.09- 0.16)	0.03 (-0.09- 0.16)	0.03 (-0.09- 0.16)	0.03 (-0.09- 0.16)
10-20 hours	0.08 (-0.00- 0.16)	0.08 (0.00- 0.16)	0.08 (0.00- 0.16)	0.08 (0.00- 0.16)	0.05 (-0.04- 0.13)	0.05 (-0.04- 0.13)	0.05 (-0.04- 0.13)	0.05 (-0.04- 0.13)
20-30 hours	0.07 (-0.02- 0.15)	0.07 (-0.02- 0.15)	0.07 (-0.02- 0.15)	0.07 (-0.02- 0.15)	0.04 (-0.04- 0.13)	0.04 (-0.04- 0.13)	0.04 (-0.04- 0.13)	0.04 (-0.04- 0.13)
30-40 hours	0.11 (0.02- 0.19)	0.11 (0.02- 0.19)	0.11 (0.02- 0.19)	0.11 (0.02- 0.19)	0.05 (-0.03- 0.13)	0.05 (-0.03- 0.13)	0.05 (-0.03- 0.13)	0.05 (-0.03- 0.13)

40+ hours	0.07 (-0.03-0.17)	0.07 (-0.03-0.17)	0.07 (-0.03-0.17)	0.07 (-0.03-0.17)	0.05 (-0.04-0.15)	0.05 (-0.04-0.15)	0.05 (-0.04-0.15)	0.05 (-0.04-0.15)
Effect on SDQ at age 14 years								
0	--	--	--	--	--	--	--	--
1-10 hours	0.33 (-0.81-1.46)	0.48 (-0.66-1.62)	0.73 (-0.38-1.84)	0.74 (-0.36-1.83)	-0.42 (-1.62-0.79)	-0.41 (-1.61-0.78)	-0.04 (-1.19-1.11)	-0.12 (-1.26-1.03)
10-20 hours	0.28 (-0.46-1.02)	0.39 (-0.35-1.13)	0.48 (-0.25-1.20)	0.51 (-0.21-1.22)	-0.14 (-0.93-0.64)	-0.16 (-0.94-0.62)	0.00 (-0.76-0.75)	0.01 (-0.74-0.75)
20-30 hours	0.07 (-0.69-0.84)	0.19 (-0.57-0.96)	0.36 (-0,39-1.11)	0.38 (-0.36-1.11)	-0.40 (-1.21-0.41)	-0.42 /-1.23-0.39)	-0.14 (-0.92-0.64)	-0.16 (-0.92-0.62)
30-40 hours	0.33 (-0.45-1.10)	0.44 (-0.34-1.21)	0.60 (-0.16-1.36)	0.62 (-0.13-1.37)	-0.26 (-1.09-0.56)	-0.29 (-1.11-0.53)	-0.01 (-0.80-0.78)	-0.04 (-0.82-0.74)
40+ hours	0.23 (-0.63-1.10)	0.36 (-0.51-1.23)	0.64 (-0.21-1.49)	0.67 (-0.17-1.51)	0.02 (-0.90-0.94)	0.03 (-0.88-0.95)	0.53 (-0.36-1.41)	0.47 (-0.41-1.35)
